# Glutamate Receptor Gene *GRIN2A*, Coffee, and Parkinson Disease

**DOI:** 10.1371/journal.pgen.1004774

**Published:** 2014-11-20

**Authors:** Taye H. Hamza, Erin M. Hill-Burns, William K. Scott, Jeffrey M. Vance, Stewart A. Factor, Cyrus P. Zabetian, Haydeh Payami

**Affiliations:** 1 Division of Genetics, Wadsworth Center, New York State Department of Health, Albany, New York, United States of America; 2 New England Research Institutes Inc., Watertown, Massachusetts, United States of America; 3 Dr. John T. Macdonald Foundation Department of Human Genetics and John P. Hussman Institute for Human Genomics, University of Miami Miller School of Medicine, Miami, Florida, United States of America; 4 Department of Neurology, Emory University School of Medicine, Atlanta, Georgia, United States of America; 5 VA Puget Sound Health Care System and Department of Neurology, University of Washington, Seattle, Washington, United States of America; 6 Department of Biomedical Science, School of Public Health, State University of New York, Albany, New York, United States of America; The Wellcome Trust Centre for Human Genetics, University of Oxford, United Kingdom

## Overview

Epidemiological studies have firmly established that coffee drinking is inversely associated with risk of developing Parkinson disease (PD) [1], and clinical trials of caffeine for treatment of PD have shown symptomatic benefit [2]–[4]. Identifying genes that modulate the efficacy of coffee may therefore have pharmacogenomic potential for prevention and treatment.

The first evidence for involvement of glutamate receptor gene *GRIN2A* came from a genome-wide association and interaction study (GWAIS) that suggested that the protective effect of coffee against PD is stronger in carriers of *GRIN2A* rs4998386_T allele than in CC homozygotes [5]. The GWAIS was conducted using the NeuroGenetics Research Consortium (NGRC) dataset, which is a single dataset of 1,458 cases and 931 controls with complete genotype and exposure data collected using NGRC's uniform study design and protocols. The finding was replicated with statistical significance in the PAGE dataset (Parkinson's, Genes, and Environment from the prospective National Institutes of Health [NIH]-AARP Diet and Health Study cohort [6]), which is also a single uniformly collected dataset of 525 cases and 1,474 controls. Two smaller datasets, PEG (Parkinson, Environment, and Gene [7]), with 280 cases and 310 controls, and HIHG (Hussman Institute for Human Genomics [8]), with 209 cases and 133 controls, also yielded results consistent with GWAIS.

Yamada-Fowler et al. [9] have independently replicated the GWAIS finding in an ethnically homogenous population in southern Sweden with significant evidence for *GRIN2A*–caffeine interaction on PD risk (P<0.001).

In this issue of *PLOS Genetics*, Ahmed et al. [10] report a pooled analysis of four datasets (Rochester, Seattle, France, and Denmark). Rochester (315 discordant sibling pairs) and Seattle (386 cases and 502 controls) did not show the expected significant inverse association between coffee and PD. France (300 cases and 598 controls), exhibiting a modest (P<0.05) coffee–PD association, showed a stronger inverse association between coffee and PD in *GRIN2A* rs4998386_T allele carriers than in CC homozygotes, a trend consistent with prior studies but not statistically significant. Denmark (1,288 cases and 1,394 controls) had by far the largest dataset and the strongest PD–coffee association (P<0.001), and it confirmed *GRIN2A*–coffee interaction with statistical significance (one-sided P_Interaction_ = 0.04) in the same direction as GWAIS: inverse association of coffee with PD was significantly stronger in *GRIN2A* rs4998386_T allele carriers (OR = 0.42/3.88 = 0.11) than in CC homozygotes (OR = 0.50).

The *GRIN2A* variants are noncoding, which raises the question of whether they are involved in gene regulation. Since the original publication of GWAIS, Grundberg et al. [11] have released genome-wide association results for methylation quantitative trait loci (mQTL). Housed on the Genevar website (http://www.sanger.ac.uk/resources/software/genevar/), these data enable investigators free access to their test results on association of single nucleotide polymorphisms (SNPs) with methylation patterns of neighboring genes. We take this opportunity to report that SNPs from the *GRIN2A–*coffee interaction peak are associated with methylation of the *GRIN2A* gene, with the significance values reaching P = 10^−7^.

To summarize, there are currently nine datasets in the literature that have examined PD risk as a function of *GRIN2A* and coffee use (classified as ever/never or by dose): four have statistically significant evidence in favor of *GRIN2A* being a modifier of PD–coffee association (NGRC, PAGE, Sweden, and Denmark) and three show a consistent trend towards interaction (HIHG, PEG, and France). In addition, there is functional evidence that the *GRIN2A* markers influence *GRIN2A* gene regulation.

## Response to Ahmed et al

In this issue of *PLOS Genetics*, Ahmed et al. dispute the *GRIN2A* effect with a paper entitled “Lack of Replication of the *GRIN2A*-by-Coffee Interaction in Parkinson Disease” [10]. There are too many fundamental differences between our study and Ahmed et al.'s to attempt a direct replication: they differ in exposure (participants in the Danish study drink twice as much coffee as those in NGRC, PEG, and others), study design (the Rochester study is based on discordant sib-pairs), the primary choice of exposure classification (high/low versus never/ever), presence of the inverse association of PD with coffee (not found in two of Ahmed et al.'s datasets), and analytic approach (the extent of testing for heterogeneity). That they did not replicate the exact parameters of GWAIS is not surprising. That they found the same trend in at least two datasets is encouraging.

The four datasets used by Ahmed et al. were heterogeneous and should not have been pooled. In our original publication [5], we examined our datasets for heterogeneity with respect to several important variables: rs4998386 allele frequency, coffee use, family history of PD, and age at onset of PD symptoms. Ahmed et al. examined only the allele frequency. Based on the raw data provided in Ahmed et al.'s Table S4, panel 1, the degree of heterogeneity in the association of coffee with PD exceeded the acceptable levels for pooling (P = 0.01). Even when they excluded Rochester (sib-pairs), the heterogeneity across France, Denmark, and Seattle was too high for pooling (P = 0.005). Pooling heterogeneous datasets can mask effects that are present in some but not other datasets.

The (or “a”) source of heterogeneity is spelled out in the text: the Rochester and Seattle datasets did not have the significant inverse association of coffee with PD that was found in the French dataset and, more remarkably, in the Danish dataset. Denmark, with the largest dataset (N = 2,682) and the strongest evidence for the inverse association of PD with caffeine (P<0.001 Table S2 of Ahmed et al. [10]), was the most powerful of the four datasets for Ahmed et al. to have asked whether genotype plays a role in PD–coffee association. In Table S5, Ahmed et al. show that the inverse association of PD with coffee was in fact significantly stronger in rs4998386_T carriers (OR = 0.42/3.88 = 0.11) than in CC homozygotes (OR = 0.50); P_Interaction_ (one-sided OR_CT+TT_<OR_CC_) = 0.04, thus replicating the proposed interaction. France (N = 898), with only one-third the sample size of Denmark and weaker coffee–PD association (P<0.05, Table S2 of Ahmed et al. [10]), had less analytic power than Denmark. As expected, the results for France were consistent but not statistically significant: as reported in Table S5 of Ahmed et al. [10], the inverse association of coffee with PD was stronger in CT+TT (OR = 0.79/1.25 = 0.63) than in CC (OR = 0.75), P_Interaction_(one-sided OR_CT+TT_<OR_CC_) = 0.34.

We would like to address another issue raised by Ahmed et al.: namely, the association of *GRIN2A* with coffee in controls. Ahmed et al. “reanalyzed” our data and noted that *GRIN2A*_T allele is positively associated with coffee drinking in controls and suggested that “the original finding may have been driven by an association of coffee consumption with rs4998386 in controls.” It is not clear why association of rs4998386 and coffee in controls is offered as an argument against an interaction between these two factors. The analysis used was case control, which does not require the independence of genotype and exposure in controls (unlike a case-only analysis), and the pattern observed may very well fit a biologically plausible model of interaction: two interacting factors inversely associated with risk of developing disease would likely be positively associated with each other in disease-free controls. *GRIN2A*_T allele was indeed more prevalent among heavy coffee drinkers in our control group, but importantly, it was not associated with heavy coffee drinking in cases. The same appears to be true in the Danish dataset: although Ahmed et al. state that “none of the datasets analyzed here indicated an association between rs4998386 and coffee consumption among controls,” the data shown in their Table S4 show a strong positive association between rs4998386 and coffee drinking in Danish controls (OR = 5.07, P = 0.03) but not in Danish cases (OR = 1.16, P = 0.63). This is interaction. As Ahmed et al. point out, *GRIN2A*_T is not associated with coffee use in the general population, yet interestingly, it is strongly associated with coffee use in control populations that excluded individuals who had PD. These observations are not inconsistent with a synergistic interaction between *GRIN2A* and coffee in reducing PD risk.

In conclusion, Ahmed et al.'s data can be interpreted in opposite directions, depending on the investigator's choice of analytic approach, mainly regarding whether to ignore heterogeneity or to test and address it. From our lens, which is admittedly colored with the hope of finding a pharmacogenomic marker to enable clinical trials for drug discovery, Ahmed et al.'s results are encouraging. We will not know for sure if *GRIN2A* is a useful pharmacogenomic marker until it is tested in clinical trials. Before formulating a clear hypothesis for pharmacogenomic trials, there is still an important issue that needs to be resolved, namely, obtaining a better understanding of the heterogeneity in the inverse association of coffee with PD and determining how best to classify coffee intake in any given cohort to maximize the power of detecting gene–drug interaction.
